# Bioactive poly (methyl methacrylate) bone cement for the treatment of osteoporotic vertebral compression fractures

**DOI:** 10.7150/thno.44428

**Published:** 2020-05-17

**Authors:** Jinjin Zhu, Shuhui Yang, Kaiwen Cai, Shuo Wang, Zhiye Qiu, Junfei Huang, Guoqiang Jiang, Xiumei Wang, Xiangqian Fang

**Affiliations:** 1 State Key Laboratory of New Ceramics and Fine Processing, School of Materials Science and Engineering, Tsinghua University, Beijing 100084, China; 2 Department of Orthopaedic Surgery, Sir Run Run Shaw Hospital, Zhejiang University School of Medicine & Key Laboratory of Musculoskeletal System Degeneration and Regeneration Translational Research of Zhejiang, Hangzhou 310016, China; 3 Department of Spinal Surgery, The Affiliated Hospital of Medical School of Ningbo University, Ningbo 315020, China; 4 Shimadzu (China) Co., Ltd. Shenzhen Branch, Shenzhen 518042, China

**Keywords:** osteoporosis, vertebroplasty, balloon kyphoplasty, poly (methyl methacrylate) bone cement, mineralized collagen

## Abstract

**Rationale:** Poly (methyl methacrylate) (PMMA) bone cement is one of the most commonly used biomaterials for augmenting/stabilizing osteoporosis-induced vertebral compression fractures (OVCFs), such as percutaneous vertebroplasty (PVP) and balloon kyphoplasty (BKP). However, its clinical applications are limited by its poor performance in high compressive modulus and weak bonding to bone. To address these issues, a bioactive composite bone cement was developed for the treatment of osteoporotic vertebral compression fractures, in which mineralized collagen (MC) was incorporated into the PMMA bone cement (MC-PMMA).

**Methods:** The *in vitro* properties of PMMA and MC-PMMA composite bone cement were determined, including setting time, compressive modulus, adherence, proliferation, and osteogenic differentiation of rat bone mesenchymal stem cells. The *in vivo* properties of both cements were evaluated in an animal study (36 osteoporotic New Zealand female rabbits divided equally between the two bone cement groups; PVP at L5) and a small-scale and short-term clinical study (12 patients in each of the two bone cement groups; follow-up: 2 years).

**Results:** In terms of value for PMMA bone cement, the handling properties of MC-PMMA bone cement were not significantly different. However, both compressive strength and compressive modulus were found to be significantly lower. In the rabbit model study, at 8 and 12 weeks post-surgery, bone regeneration was more significant in MC-PMMA bone cement (cortical bone thickness, osteoblast area, new bone area, and bone ingrowth %; each significantly higher). In the clinical study, at a follow-up of 2 years, both the Visual Analogue Score and Oswestry Disability Index were significantly reduced when MC-PMMA cement was used.

**Conclusions:** MC-PMMA bone cement demonstrated good adaptive mechanical properties and biocompatibility and may be a promising alternative to commercial PMMA bone cements for the treatment of osteoporotic vertebral fractures in clinical settings. While the present results for MC-PMMA bone cement are encouraging, further study of this cement is needed to explore its viability as an ideal alternative for use in PVP and BKP.

## Introduction

Osteoporosis, which is accompanied by chronic bone pain and muscle weakness, affects 200 million people worldwide, particularly post-menopausal women 1, 2. As the global population ages, the incidence of osteoporosis is increasing 3. Osteoporosis is a systemic, multifactorial disease with osteopenia and bone mass loss that can damage the microstructure of bone and increase bone fragility, rendering it susceptible to systemic fracture and osteopathy 4, 5. Osteoporotic vertebral compression fractures (OVCFs) are common in osteoporosis 6. Conservative methods to treat the pain resulting from OVCFs, such as oral medications and immobilization, have limitations in their ability to rapidly cure and relieve patient suffering 7, 8. Therefore, vertebral body augmentation/stabilization procedures, commonly percutaneous vertebroplasty (PVP) and balloon kyphoplasty (BKP), are also commonly used. Various bone cements are used in these procedures, with the most common being poly (methyl methacrylate) (PMMA) 9. Plain PMMA bone cement exhibits significantly higher stiffness than the surrounding trabecular bone (E_cement_ = 1700-3700 MPa; E_bone_ = 10-900 MPa 10), and this difference contributes to an increased risk of fractures in the adjacent vertebrae and the collapse of augmented vertebrae 11, 12. In addition, PMMA possesses inferior bioactivity and cannot be absorbed and replaced by new bone tissue. Thus, the boundaries between bone and PMMA are still distinct after several years *in vivo* 13, 14, which can result in the loosening or dislodgement of the bone cement 15. Consequently, some patients require further surgical treatment. To overcome these deficiencies, modifications of PMMA-based bone cement have been studied extensively. Various approaches have been employed to modify PMMA-based materials in recent decades 16, including natural bone powder hydroxyapatite (HA) 17, linoleic acid (LA) 18, chitosan 19, 20, and small intestinal submucosa 21. However, most of these methods lead to problems, due to the material not meeting the surgical requirements or having a compressive strength that is too low 22, 23.

Mineralized collagen (MC) 24, which is composed of organic type-I collagen and nano-hydroxyapatite (nHA) and simulates the chemical composition and microstructure of natural bone matrices, was previously developed in our laboratory via *in vitro* biomimetic mineralization. Among them, HA nanocrystals grew periodically and orderly on the surface of collagen fibers, with their crystallographic c-axes preferentially aligned parallel to the long axis of the collagen fibers 25, 26. In previous studies, we have indicated that MC has very good biocompatibility and osteogenic abilities, and as such has been widely used in the repair of diverse bone defects, as well as for promoting *in situ* bone regeneration 27. In addition, MC has been proposed for the modification of PMMA bone cement. The optimal proportion and size of MC particles have been investigated in detail to ensure appropriate injectability and mechanical properties for use in clinical applications 28. The modified MC-PMMA bone cement has previously exhibited very good biosafety in a rabbit joint defect model 29. Therefore, we believe that MC-PMMA could have great potential for use in the repair of OVCFs in clinical settings. The preliminary clinical assessment indicated its feasibility for OVCFs with acceptable leakage behavior and clinical recognition 15, 30, 31. Consequently, it is of vital importance to investigate the bioactivity and effectiveness of MC-PMMA bone cement for vertebral repair with a broad spectrum of assessments from *in vitro* to *in vivo*.

Therefore, the purpose of the present study was to systematically evaluate the optimized bioactive MC-PMMA bone cement for the treatment of osteoporotic vertebral compression fractures in specific clinical applications (PVP and BKP). Here, the *in vitro* properties, including setting time compressive modulus, and biocompatibility measures (adhesion, proliferation, and differentiation of rat bone marrow-derived mesenchymal stem cells) of PMMA and MC-PMMA bone cements were evaluated. Then, an osteoporotic New Zealand female rabbit model was developed and used to compare the augmentation/stabilization effects of these two cements when used after PVP. Finally, a small clinical study was conducted to compare the performance and repair effects of augmenting OVCFs.

## Materials and Methods

### Study design

**Scheme 1:**
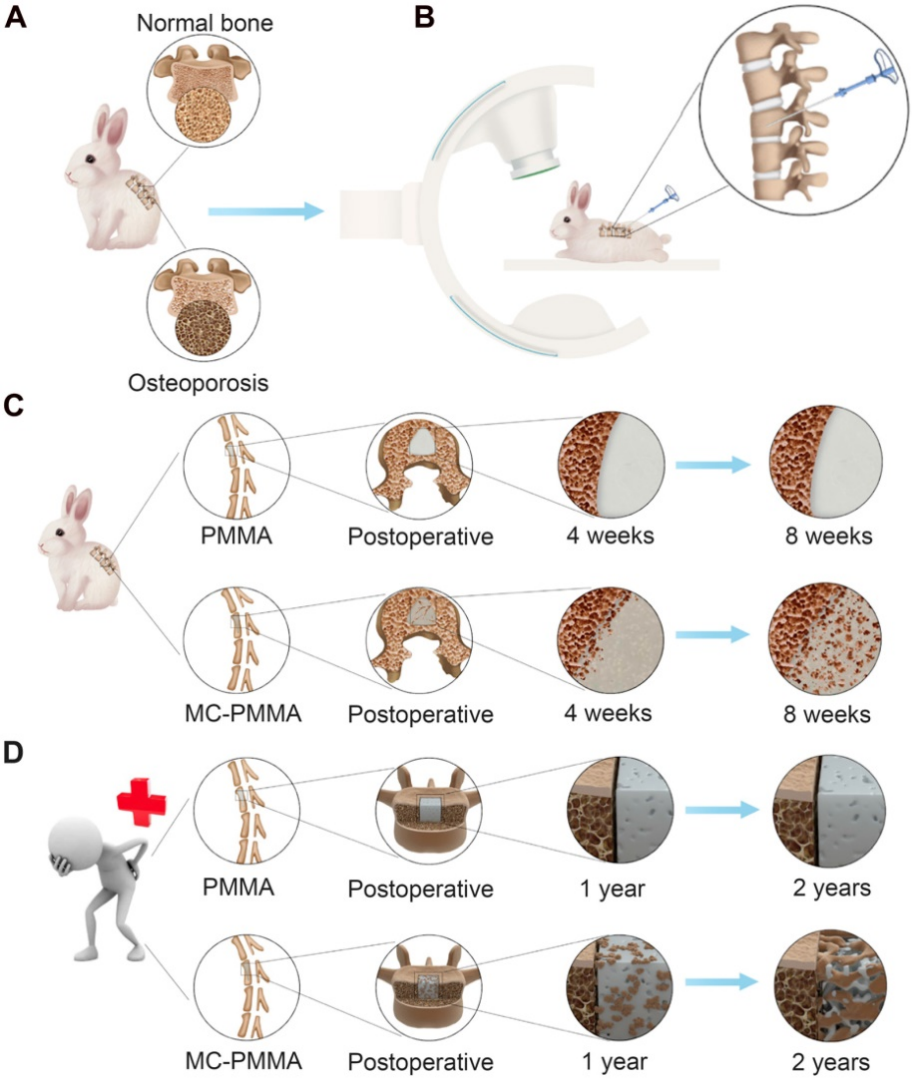
Study design and bone regeneration on the interface between bone and bone cement. (A) Osteoporotic rabbit model. (B) PVP in osteoporotic rabbit model. Bone growth features of PMMA bone cement and MC-PMMA bone cement in rabbits (C) and human body (D).

A schematic drawing of the main elements of the osteoporotic rabbit model and the clinical study are presented in Scheme 1.

### Synthesis of MC and preparation of bone cements

**Figure 1:**
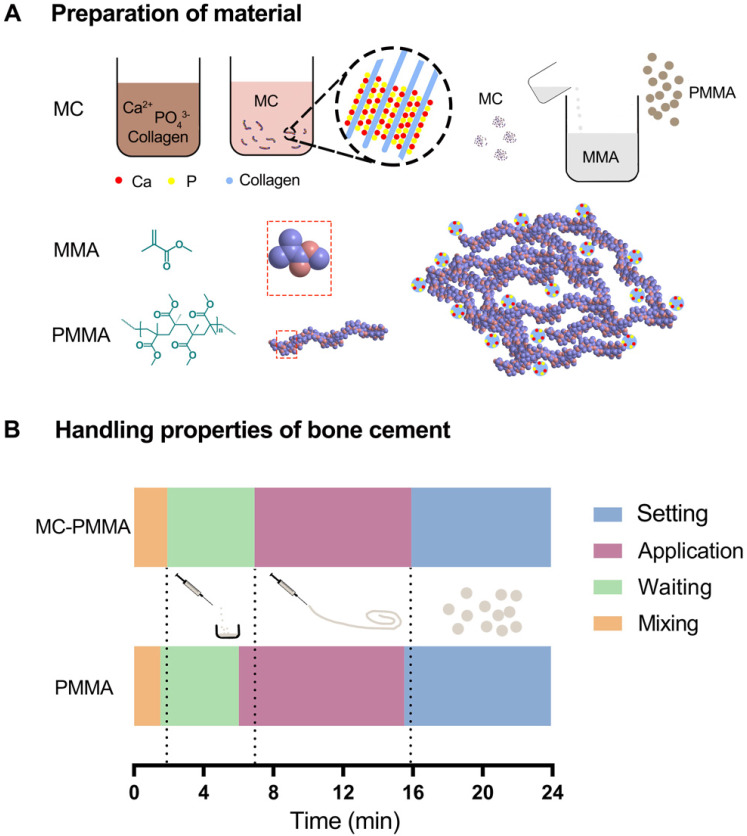
(A) Preparation of mineralized collagen and PMMA bone cement. (B) The handling properties of bone cements.

MC powder was prepared as previously described 25. Soluble calcium and phosphate were added to an acidic collagen solution and mixed. Sodium hydroxide solution was slowly added dropwise to increase the pH, which caused the calcium and phosphate ions to nucleate and grow into nano-hydroxyapatite arranged along the collagen fibers (i.e. MC). When the pH of the mixture approached neutral, the MC was precipitated. The resulting MC was washed with water and lyophilized for 48 h. After grinding, it was passed through 200-300 mesh screens to obtain MC powder, as shown in Figure 1A.

PMMA bone cement (Mendec Spine Cement; Tecres SPA, Verona, Italy) is commercially available and has been approved for use in PVP and BKP. The compositions of PMMA and MC-PMMA bone cements are presented in Table 1. The MC-PMMA bone cement was prepared by mixing the MC particles with PMMA powder, followed by the addition of liquid MMA to the PMMA and MC powder mixture (Figure 1A). The optimal mix ratio of MC particles was 15% by weight, as evaluated in our previous study 28.

For each bone cement, the powder and liquid (in a ratio of 2.26/2.56 g mL^-1^) were thoroughly mixed to obtain homogeneous dough, which was then injected into a stainless steel mold (Φ13 mm × 1 mm). After curing for 24 h at 37°C in 5% CO_2_, the cylindrical piece was removed from the mold, cleaned with medical alcohol, dried in air, and then sterilized using 15 kGy gamma-ray irradiation. The material used for the animal models was prepared during surgery; the MMA monomer liquid and the powders were mixed until reaching the viscous wire drawing stage, after which the cement was injected.

### Molecular structures and internal and external surface morphologies

PMMA and MC-PMMA were broken into smaller, uniform pieces. The sample preparation steps were as follows: crush the material, pass a 200-300 mesh inspection sieve, and place the fine powder in a 37°C constant-temperature drying oven to constant weight, mix with appropriate KBr, grind, and press. The infrared absorption spectrum of the sample was measured within a wavenumber range of 400-4000 cm^-1^.Their molecular structure was characterized using a Thermal Analysis Hybrid System (TG-MS-FTIR-X70; NETZSCH Groups, Selb, Germany).

The internal and external morphologies of the prepared cement discs were obtained using a scanning electron microscope (SEM) (GEMINISEM 500; Carl Zeiss, Jena, Germany). To observe the external morphology, a prepared cement disc was bonded to the surface of the aluminum sample table using an adhesive strip. To observe the internal morphology, a prepared cement disc was broken (break the above densified and mineralized collagen disc, select a flat section, and use a conductive adhesive to stick it on the side of the aluminum sample table). The flat cross-sections were selected and bonded to the side of the aluminum sample table with an adhesive strip. A disc or a flat disc piece was sprayed with gold for 90 s and placed on the SEM sample table. The sample chamber was placed under vacuum, and the sample was observed. A rectangular region of ~400 μm^2^ was selected randomly, and the proportions of Ca and P were determined by energy dispersive X-ray spectrometry.

### Handling times

The following handling times were determined: mixing time, waiting time, working time, and setting time. Mixing time was the time taken for the complete mixing of the powder and liquid constituents of the cement; waiting time (or so-called “toothpaste period”) was the time required for the vertical push of bone cement to extrude 1 cm higher than the push rod opening; working time (or application time) was the time when the bone cement was suitable for pushing into the fractured vertebral body, and setting time was the time taken for the cement to solidify to the point where it could be extruded from the mold intact.

### Compressive properties

The homogeneous cement dough was poured into a stainless steel die to produce cylindrical specimens (Φ 6 mm × 12 mm, n=6). The compressive strength and modulus were determined using a universal materials testing machine (AG-X100KN; Shimadzu, Kyoto, Japan), operated at a cross-head speed of 20 mm/min.

### *In vitro* culture of bone mesenchymal stem cells

Rat bone mesenchymal stem cells (BMSCs) (cat. no. RASMX-90011; Cyagen Biosciences, Inc., CA, USA) were seeded on the surface of bone cement at a density of 6 × 10^3^ cells/cm^2^ (Φ 13 mm × 1 mm, n=5). The culture medium (cat. no. RASMD-90011; Cyagen Biosciences, Inc., CA, USA) supplemented with 10% fetal bovine serum (FBS) (Gibco, Carlsbad, CA, USA) was changed after 24 h and every 3 days thereafter. The cells were cultured at 37°C in a humidified atmosphere containing 5% CO_2_ (MCO-15AC; Sanyo, Osaka, Japan). On days 1, 3, 5, and 7, 200 μL of Cell Counting Kit-8 (CCK-8) (Dojindo, Tokyo, Japan) working solution was added to the culture medium and incubated for 2 h at 37°C in the dark. Then, 100 μL of the supernatant was transferred to a 96-well plate, and the absorbance at 450 nm was measured using a Multimode Plate Reader (iMark™ 168; Bio-Rad Laboratories, Inc., California, USA).

BMSCs were seeded onto bone cement in 24-well cell culture plates at 1.5 × 105 cells per well (Φ 13 mm × 1 mm, n=5). The cells were cultured at 37°C in a humidified atmosphere containing 5% CO_2_. When the cells reached ~70% confluence, the medium was changed to osteogenic induction medium (cat. no. RASMX-90021; Cyagen Biosciences Inc., CA, USA) and the cells were cultured for another two weeks. During incubation, half of the culture medium was replaced every 3 days.

### Immunofluorescence staining

In order to observe the adhesion behavior of the cells on bone cement, cytoskeletal staining was performed after 1, 3, 5, and 7 days in the proliferation experiment and 14 days after changing to osteogenic induction medium in the differentiation experiment. Briefly, the cells were gently washed with phosphate-buffered saline (PBS) (R21-040-CV; Corning, New York, USA) three times after removing the culture medium, and were then fixed with 4% paraformaldehyde (BL539A; Saiguo Biotech Co., Guangzhou, China) for 30 min. After washing with PBS three times, the cells were treated with 0.1% Triton (X-100; Cell Signaling Technology, MA, USA) in PBS for 5 min, followed by 1% bovine serum albumin (BSA) (Cell Signaling Technology, MA, USA) in PBS for 30 min at 37°C. After the removal of this solution, the cells were stained with rhodamine-phalloidin (1:300) (cat. no.PHDR1; Cytoskeleton, Denver, CO, USA) at 37°C for 40 min and washed with PBS. After staining with 4', 6-diamidino-2-phenylindole (DAPI) (D3571; Life Technologies, Massachusetts, USA) for 10 min and washing with PBS, the samples were observed under laser confocal microscopy (LSM710; Carl Zeiss, Jena, Germany). The adherent cell areas and pseudopod lengths were quantified using Image Pro (Image Pro-Plus 6.0; Media Cybernetics Inc., MD, USA) (n=6).

### Morphology of BMSCs

The morphology of the BMSCs was observed by SEM. After 12 h, the medium was removed, and 500 μL of 2.5% glutaraldehyde was added to each well. The samples were placed at room temperature for 2 h. Gradient dehydration was carried out successively in 30%, 50%, 70%, 80%, 90%, and 95% ethanol, followed by 100% anhydrous ethanol, for 30 min per step. Afterward, the samples were immersed in tert-butanol for 48 h and freeze-dried for SEM observation.

### Quantitative alkaline phosphatase activity assay

On day 14, after the removal of the culture medium, the cells (n=5) were washed twice with pre-cooled PBS, and 100 μL of radioimmunoprecipitation assay (RIPA) (Beyotime, Shanghai, China) lysis buffer was added to each sample and incubated for 15 min on ice. The supernatants were assayed for ALP activity, which was normalized to total protein content measured using a bicinchoninic acid (BCA) kit (Beyotime, Shanghai, China).

### Quantitative reverse transcription polymerase chain reaction (RT-qPCR)

After 14 days of culture, total RNA (n=5) was extracted using the mRNA Isolation Kit (DP501; Tiangen, Beijing, China) and quantified using a Nucleic Acid and Protein Analyzer (Microfuge18; Beckman-Coulter, California, USA). The RNA was reverse transcribed to cDNA using the FastQuant RT Kit (KR-106; Tiangen, Beijing, China). The corresponding primer sequences are listed in Table S1. qPCR was performed using the iTaq Universal SYBR Green Supermix (172-5122; Bio-Rad Laboratories, Inc. California, USA).

### Preparation of the rabbit osteoporosis model

All animals were treated and raised according to the standard guidelines, approved by the Institutional Animal Ethics and Welfare Committee of Ningbo University (2016-15). Forty-two 5-month-old New Zealand female rabbits, weighing 3.02 ± 0.25 kg, were purchased from Yanling Rex Rabbit Farm in Ningbo and raised by the Animal Laboratory Center of the Medical School of Ningbo University for two weeks with adaptive feeding. The bone mineral density (BMD) of the lumbar spine was measured using dual-energy X-ray (DISCOVERY CT750 HD; General Electric Company, New York, USA) one week before surgery. All animals were randomly divided into osteoporosis (castration + hormone, n=39) and normal (sham operation + saline, n=3) groups. After 12 h of fasting, the rabbits were anesthetized by injecting 3% pentobarbital sodium (30 mg/kg) into the ear vein, and the skin was exposed adequately. Under aseptic conditions, the animals were secured on the operating table in the prone position and the skin was disinfected with iodophor solution. For the osteoporosis group, ovariectomy was performed through the dorsal side, while the normal group was subjected to all surgical procedures, except ovary removal. After surgery, 20 IU/kg/day penicillin was routinely used, and all animals were fed similarly. For the osteoporosis group, dexamethasone sodium phosphate was injected intramuscularly at 0.6 mg/kg/day one week later for 8 weeks. Animals in the normal group were administered the same volume of saline. The BMD of the lumbar spine was measured preoperatively and at 4 and 8 weeks after surgery. Three animals were randomly selected and euthanized after 8 weeks in each group, and the attachments of the vertebral femur were removed.

### Osteoporotic rabbit model

Thirty-six osteoporotic New Zealand female rabbits in the osteoporosis group were divided randomly into two equal-sized groups: the PMMA and MC-PMMA bone cement groups. After a 12-h fast, the rabbits were anesthetized using 3% pentobarbital (30 mg/kg) administered through the ear vein, placed in the prone position, and routinely disinfected. Injection points were selected by inserting a needle hole near the needlepoint of the vertebral body. The needle holes were inserted directly after the needle hole size incision was made near the needlepoint of the vertebral body. The L5 spinous process was confirmed by palpation, and a 14G bone marrow cannula (Myelo-Gal; Iskra Industry Co. Ltd., Tokyo, Japan) was inserted into the bone point at 1-2 mm. The transverse process and vertebral body were monitored by X-ray (Toshiba X-ray machine; TOSHIBA, Tokyo, Japan). After reaching the appropriate position, the cannula was filled with material and the needle was retracted after the injection material solidified. No rabbits died during the surgery, and only a small amount of bleeding, wounds or other injuries occurred. Penicillin was administered intramuscularly 2 h before surgery and 3 days after surgery to prevent postoperative monitoring. The wound healing, mental state, activity, and diet consumption of the animals were monitored postoperatively.

### Micro-CT imaging of the treated vertebral body

The rabbits were euthanized at 4, 8, and 12 weeks after PVP (n=6), and the attachments of the vertebral femur were removed. All vertebral femoral samples were scanned by micro-CT (InspeXio SMX-225 CT FPD HR; Shimadzu Co. Ltd., Kyoto, Japan). Each sample was reconstructed using a data analyzer (VGStudio MAX; Volume Graphics, Heidelberg, Germany) under the same conditions. An industrial micro-CT system was used at 225-kV accelerating voltage and 4-μm resolution ratio to scan the vertebral body. Cylinders of 1.5 mm in diameter and 1.5 mm in height were taken from the vertebral bone and femur to determine the trabecular thickness. The reconstruction parameters of the cancellous bone analysis in all samples were as follows: gray scale of cancellous bone > 1000 and bone surface/bone volume (BS/BV) ratio = 1/mm.

### Histological staining and imaging

The rabbits were euthanized at 4, 8, and 12 weeks after PVP (n=6). Vertebral samples from the rabbits in both groups were fixed with 10% formalin and dehydrated with gradient alcohol concentrations of 70%, 80%, 90%, 95%, and 100%. Then, the samples were embedded with PMMA embedding agent and cut into sections of 10-20 μm using a diamond tissue microtome (SAT-001-E300CP; EXAKT, Hamburg, Germany). At 4, 8, and 12 weeks post-surgery, the polymer-containing vertebrae from both groups were harvested after localization with micro-CT (n=6). Then, hard tissue sections were obtained and stained with methylene blue and basic fuchsin. All of the stained slides were observed with a slide scanner (Axio-Scan Z1; Carl Zeiss, Jena, Germany). To quantify the regenerative capacity of the cement, histological scoring was performed on 7 images by three colleagues according to the guidelines in Table 2 to assess tissue response at bone-implant interfaces, new bone quality, and bone ingrowth. Cortical bone thickness, osteoblast area, new bone area, and percentage of bone growth were calculated. First, we identified the materials in the low-magnification images. Seven different areas near the interfaces of materials and the surrounding tissues from each image were selected at random. At least three images from each rabbit were randomly selected. Next, under high magnification, Image Pro Plus software was used to select the new bone tissues. The new bone was stained in purple, the mature bone in red, and the osteoblasts in blue.

### Statistical analysis

All results are presented as the mean ± standard deviation (SD). For *in vitro* studies, each experiment was conducted independently at least three times. The normality test was performed using the Kolmogorov-Smirnov test in SPSS (v.23.0; IBM Corp., Armonk, NY, USA). Statistical analysis of normally distributed data was carried out using independent t-tests or one-way analysis of variance (ANOVA). Statistical analysis of the data without normal distribution was carried out using a non-parametric method in conjunction with an appropriate post-hoc test (least significant difference). Differences were considered statistically significant when *p <* 0.05, shown as *; *p <*0.01, shown as **; or *p <*0.001, shown as ***.

## Results

### Bone cement properties

**Figure 2:**
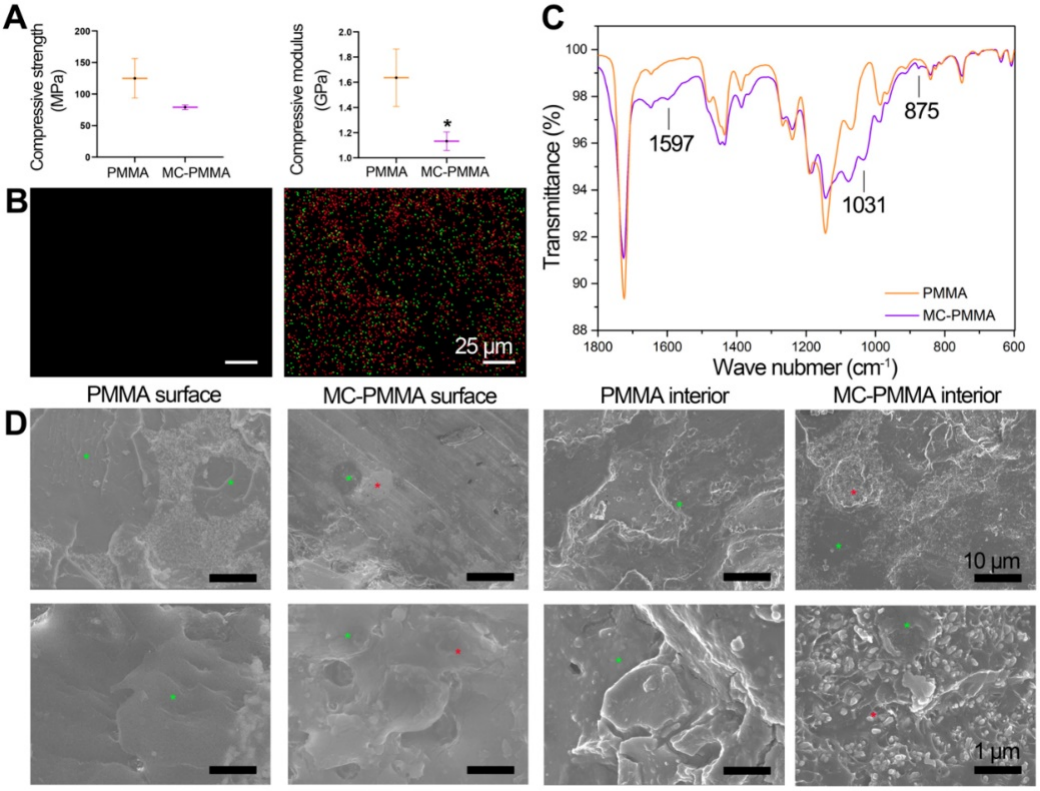
Characterization of bone cements. (A) Mechanical properties of PMMA and MC-PMMA bone cements. Results are presented as the mean ± SD; n = 5; *p < 0.05 (B) Distribution of Ca (green) and P (red) elements on MC-PMMA bone cement. (C) FTIR of PMMA and MC-PMMA bone cements. (D) SEM morphologies of superficial and internal structures of PMMA and MC-PMMA bone cements (MC labelled with * in red and PMMA labelled with * in green).

For each of the handling times, the value for the PMMA bone cement was not significantly different from the corresponding value for MC-PMMA bone cement (Figure 1B). The mixing time for PMMA and MC-PMMA was 1.5 min and 1.9 min, respectively. The application times of the PMMA and MC-PMMA bone cement were almost the same, at 9.0 and 9.5 min, respectively. Comparing the PMMA with MC-PMMA bone cement, their operational characteristics were similar, with differences of 0.5 and 1.0 min in the required time per stage (Figure 1B). The compressive strength of the MC-PMMA cement was significantly lower than that of the PMMA cement (79.12 ± 3.65 MPa versus 111.61 ± 11.43 MPa), with the same trend found for compressive modulus (1.13 ± 0.07 GPa versus 1.63 ± 0.23 GPa) (Figure 2A). Ca and P were evenly distributed in the modified PMMA bone cement, and the mole ratio of Ca to P was 1.58, which is close to the ratio in natural human bone. Apparently, Ca and P were not found in pure PMMA bone cement (Figure 2B). Fourier transform infrared spectroscopy (FTIR) spectroscopy showed the characteristic peaks of HPO_4_^2-^ at 875 cm^-1^, HPO_4_^3-^ at 1031 cm^-1^, and Ca^2+^ at 1597 cm^-1^, which verified that calcium phosphate minerals were well distributed in the modified PMMA bone cement (Figure 2C). SEM images showed that the surfaces of the PMMA and MC-PMMA bone cement were smooth and delicate. The MC particles were physically combined with PMMA without chemical interactions. The results showed that MC particles were evenly distributed on the surface and inside the MC-PMMA bone cement, and closely interconnected with PMMA without gaps; incomplete reactions and inhomogeneity of PMMA were not apparent, which indicated that the MC was compatible with the PMMA bone cement (Figure 2D). The porosity of PMMA bone cement was almost the same as that of MC-PMMA bone cement (5.61 ± 0.16% versus 7.22 ± 0.53%), as shown in Figure S1A, B, and E. Compared with the PMMA bone cement, MC-PMMA had a lower CT value (9.36 ± 0.13 versus 5.46 ± 0.22), as shown in Figure S1C, D, and F.

### Effects of bone cements on adhesion, proliferation, and differentiation behaviors of BMSCs

**Figure 3:**
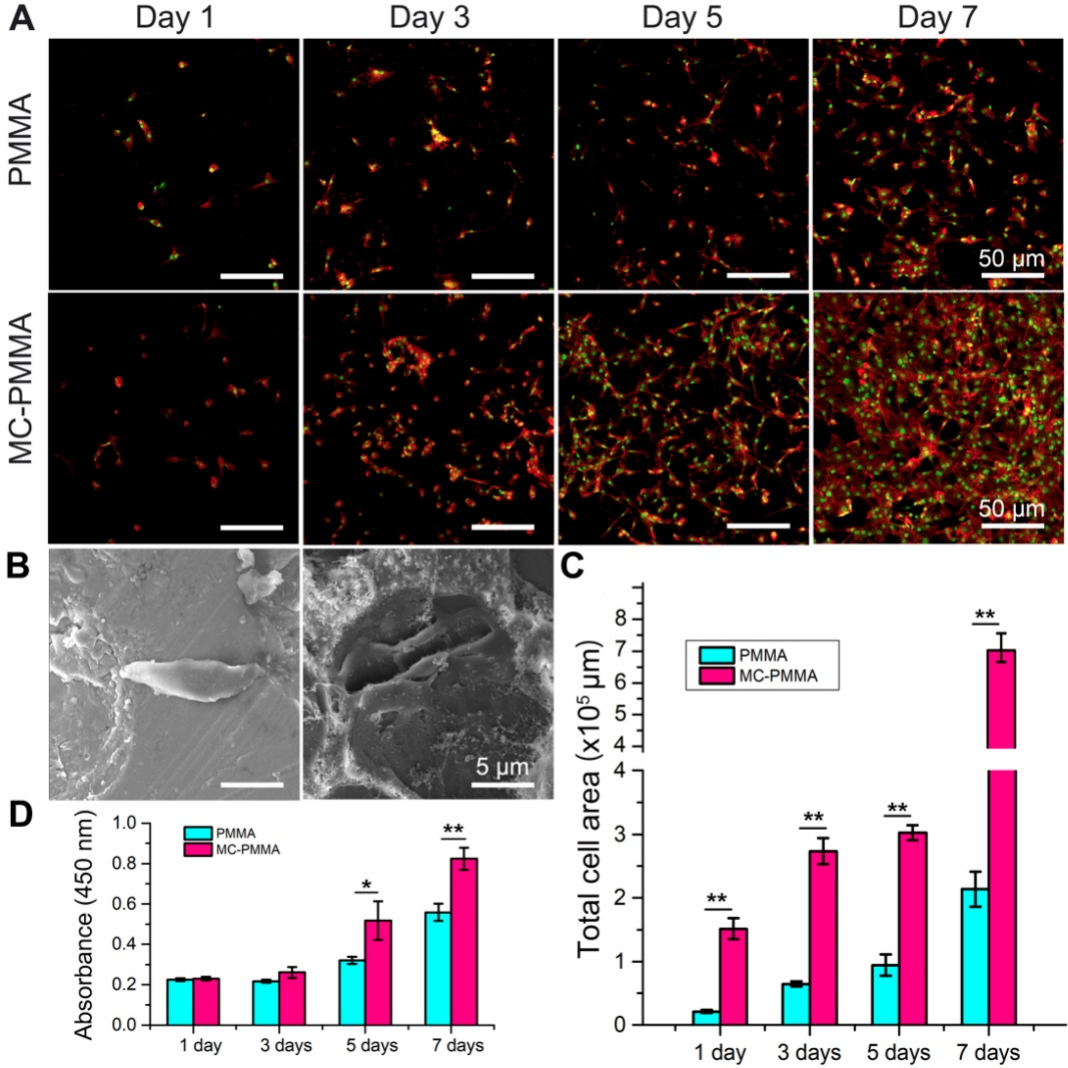
Morphology and proliferation of BMSCs on PMMA and MC-PMMA bone cements. (A) Morphology of BMSCs on days 1, 3, and 7 on MC-PMMA or PMMA bone cement. Cells were stained with Rhodamin-phalloidin for F-actin (red) and SYTOX Green for nuclei (green). (B) Cell morphology on the surface of PMMA bone cement (left) and MC-PMMA bone cement (right) observed via SEM. (C) Total cell area on MC-PMMA and PMMA bone cements. (D) Cell proliferation on day 1, 3, 5, and 7. Results are presented as the mean ± SD; n = 5; *p < 0.05 and **p < 0.01.

The cell densities at days 5 and 7 on the MC-PMMA bone cement were significantly higher than those on the PMMA bone cement (Figure 3A). BMSCs extruded more pseudopods under MC stimulation (Figure 3B). The cell spreading area on the MC-PMMA bone cement was significantly larger than that on PMMA at days 5 and 7 (Figure 3C), indicating promoted cell attachment. At day 7, the number of cells on the MC-PMMA bone cement appeared to be higher than that on the PMMA bone cement, and the spread of individual cells was better than that on PMMA. Figure 3D shows that there was a significant difference between the two groups at 5 and 7 days. After 5 days, the proliferation rate of cells on MC-PMMA bone cement was significantly higher than that on PMMA bone cement, indicating that MC-PMMA bone cement had no obvious cytotoxicity and could promote cell proliferation.

**Figure 4:**
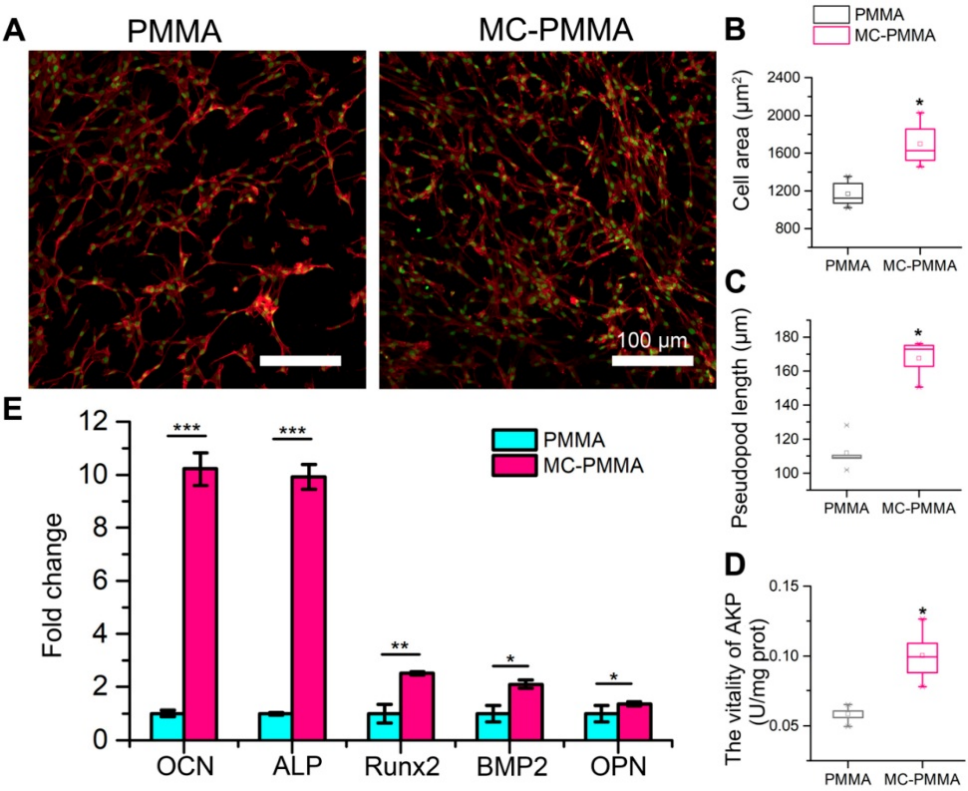
Osteogenic differentiation of BMSCs on PMMA and MC-PMMA bone cements. (A) Representative fluorescence microscopy images of BMSCs on MC-PMMA bone cement and PMMA bone cement after 14 days. Cells were stained with Rhodamin-phalloidin for F-actin (red) and SYTOX Green for nuclei (green). Cell spreading area (B) and pseudopod length (C) on MC-PMMA and PMMA bone cements. ALP activity (D) and gene expression (E) of BMSCs after 14 days. Results are presented as the mean ± SD; n = 5; *p < 0.05, **p < 0.01 and ***p < 0.001.

After 14 days of osteogenic induction, the cytoskeleton staining of differentiated cells showed that cells on the MC-PMMA bone cement had a higher density with more pseudopods extending and spreading. However, on the PMMA bone cement, the number of cells and pseudopods was lower (Figure 4A). The cell spreading area and pseudopod length on the MC-PMMA bone cement were significantly higher than that of the PMMA bone cement, indicating that MC-PMMA bone cement could promote the differentiation of BMSCs (Figure 4B‒C). Alkaline phosphatase (ALP) activity is a marker of osteogenic differentiation, which could indicate the effect of materials on promoting BMSC differentiation. A quantitative assay showed that the ALP activity of cells on MC-PMMA bone cement was significantly higher than that on PMMA bone cement after 14 days, indicating that MC could promote the osteogenic differentiation of BMSCs and induce the upregulation of ALP expression (Figure 4D). Osteocalcin (OCN), ALP, Runt-related transcription factor 2 (Runx2), bone morphogenetic protein-2 (BMP-2), and osteopontin (OPN) are the most common genes related to the differentiation of BMSCs. Compared to cells on PMMA bone cement, BMP-2 and Runx2 expression was upregulated nearly 1.5-fold after 14 days in the MC-PMMA bone cement (Figure 4E). ALP and OCN expression was upregulated nearly 10-fold, and OPN expression was also significantly upregulated after 14 days (Figure 4E). These results indicated that MC-modified PMMA bone cement was beneficial for BMSCs to differentiate into osteoblasts and, thus, had the potential to promote bone repair.

### Osteoporotic rabbit model

**Figure 5:**
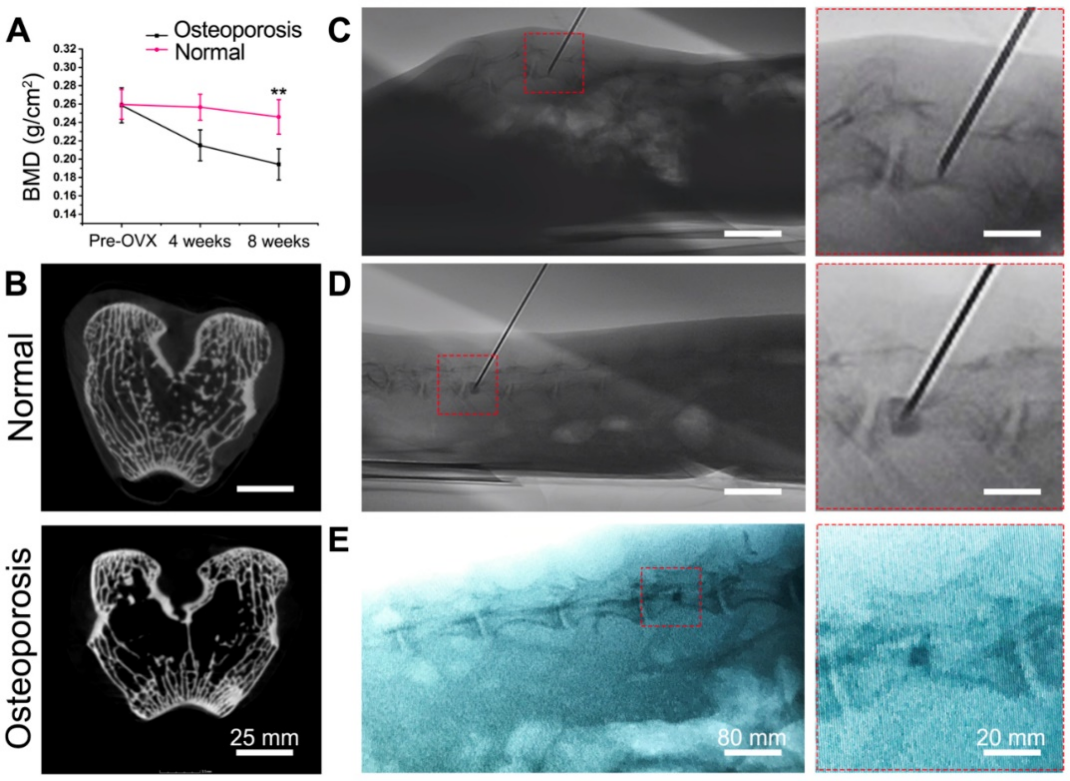
Establishment of osteoporotic rabbit model and PVP surgery. (A) Bone mineral density (BMD) of lumbar spine preoperatively, 4 and 8 weeks post-surgery. Results are presented as the mean ± SD; n = 3; **p < 0.01. (B) Micro-CT images of rabbit femur in normal group and osteoporosis group. (C, D) Intraoperative and postoperative conditions shown by C-arm fluoroscopy. (C) Intraoperative needle insertion position indicating the direction and location of the French bone marrow trocar. (D) Injection of bone cement. (E) X-ray imaging of bone cement 2 days after surgery.

Each of the animals developed slight swelling of the incision that healed well. After 2 weeks, the suture was removed, and no infection was observed. The activity and food intake of the animals were normal. There were no visible differences between the osteoporosis group and the normal group; however, the rabbits in the osteoporosis group had less hair. After 8 weeks, the rabbits in the osteoporosis group were prone to fracture with poor reactivity. The bone mineral density (BMD) results showed that osteoporosis developed in the osteoporosis group within 4 weeks after surgery and was obvious after 8 weeks. The BMD index in the osteoporosis group decreased by 19.03% after 4 weeks and 29.23% after 8 weeks compared with that in the normal group. There was no significant difference before and after surgery in the normal group (Figure 5A). The circular or elliptical trabeculae of rabbits in the normal group were regular in shape, uniform in thickness, interconnected, and consistent in color, with rare fractures and defects. In the osteoporosis group, the trabeculae of the vertebral bodies were sparser than that of the normal group, with uneven thickness, distribution, intermittent endpoints, and light staining; microfractures and bone defects had formed after fracture absorption in the osteoporosis group (Figure 5B). Successfully prepared rabbit models of osteoporosis were used for the *in vivo* evaluation of bone cements. The PMMA and MC-PMMA bone cements were injected using a 14G bone marrow cannula entering the L5 vertebral body of rabbits, which could be observed intraoperatively and postoperatively, as shown in Figure 5 C, D, and E.

**Figure 6:**
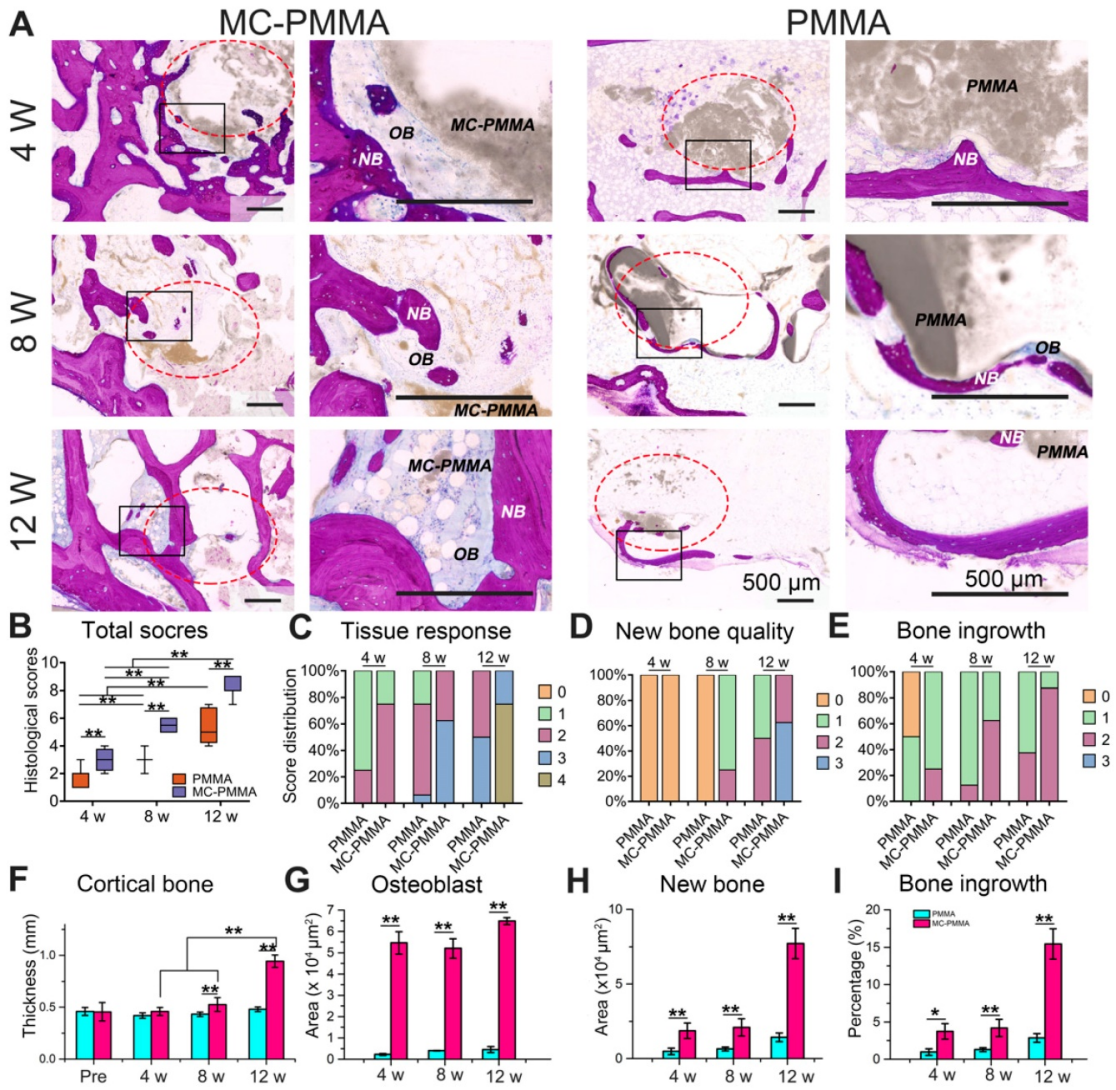
Histological analysis of bone ingrowth and regeneration. (A) Histological staining of PMMA group and MC-PMMA group after 4, 8, and 12 weeks with methylene blue (light blue) and basic fuchsin (red). The bone cement was in gray. (B) Total histological scores. Histological scores of tissue response (C), new bone quality (D), and bone ingrowth (E). (F) Cortical bone thickness (mm) preoperatively, after 4, 8 and 12 weeks. Osteoblast area (G), new bone area (H) and percentage of bone growth (I) after 4, 8 and 12 weeks. Results are presented as the mean ± SD; n = 6; *p < 0.05 and **p < 0.01.

At 4, 8, and 12 weeks, the hard tissue sections of the treated vertebra were stained with methylene blue and basic fuchsin to identify the bone repair (Figure 6A). Areas in deep purple indicated new bone tissue, while those in blue and blue-purple staining indicated osteoblasts, and bone collagen, respectively. After 4 weeks, the bone repair of the MC-PMMA group was markedly better than that of the PMMA group. At 8 weeks, the original segmented bone tissue began to connect and new bone structure began to form in the MC-PMMA group, while there was no regeneration in the PMMA group, since the boundary of bone cement was the same as that at 4 weeks. At 12 weeks, the MC in the MC-PMMA bone cement showed an apparent degradation and there was obvious bone ingrowth with osteoblasts and new bone. The boundary of PMMA bone cement was still smooth and intact with only slight bone repair. We then calculated the histological scores in each group at 4, 8, and 12 weeks according to the standard in Table 2. The total histological scores increased significantly with time in each group (Figure 6B). The total scores of the MC-PMMA group were significantly higher than that of the PMMA group at 4, 8, and 12 weeks, respectively. A histological score of 1 reflects the interaction between the tissue and bone cement. At 4 weeks, both the MC-PMMA and PMMA groups showed almost no inflammatory cells (Figure 6C). At 8 weeks, the MC degraded with apparent remodeling lacunae and osteoblast infiltration; however, the PMMA bone cement had few osteoblasts. At 12 weeks, the osteoblasts were replaced with new bone in the MC-PMMA bone cement, whereas there was little new bone in the PMMA bone cement. Histological scores of 2 and 3 indicated new bone quality and bone ingrowth (Figure 6D‒E). At 4 weeks, the specimens in both groups showed similar bone ingrowth, and there was almost no new bone or immature neonatal bone tissue. At 8 weeks, new bone was obvious and well developed in the MC-PMMA group, while little new bone was found in the PMMA group. At 12 weeks, the boundaries and some interior regions of the MC were almost completely replaced by new bone with thick cortical bone surrounded by dense trabeculae; however, the boundary of the PMMA bone cement was clearly defined at 12 weeks and there was little bone ingrowth. The cortical bone thickness, osteoblast area, new bone area, and percentage of bone growth in the MC-PMMA group at each time point were significantly higher than those in the PMMA group, which was in accordance with the histological scores (Figure 6F‒I).

**Figure 7:**
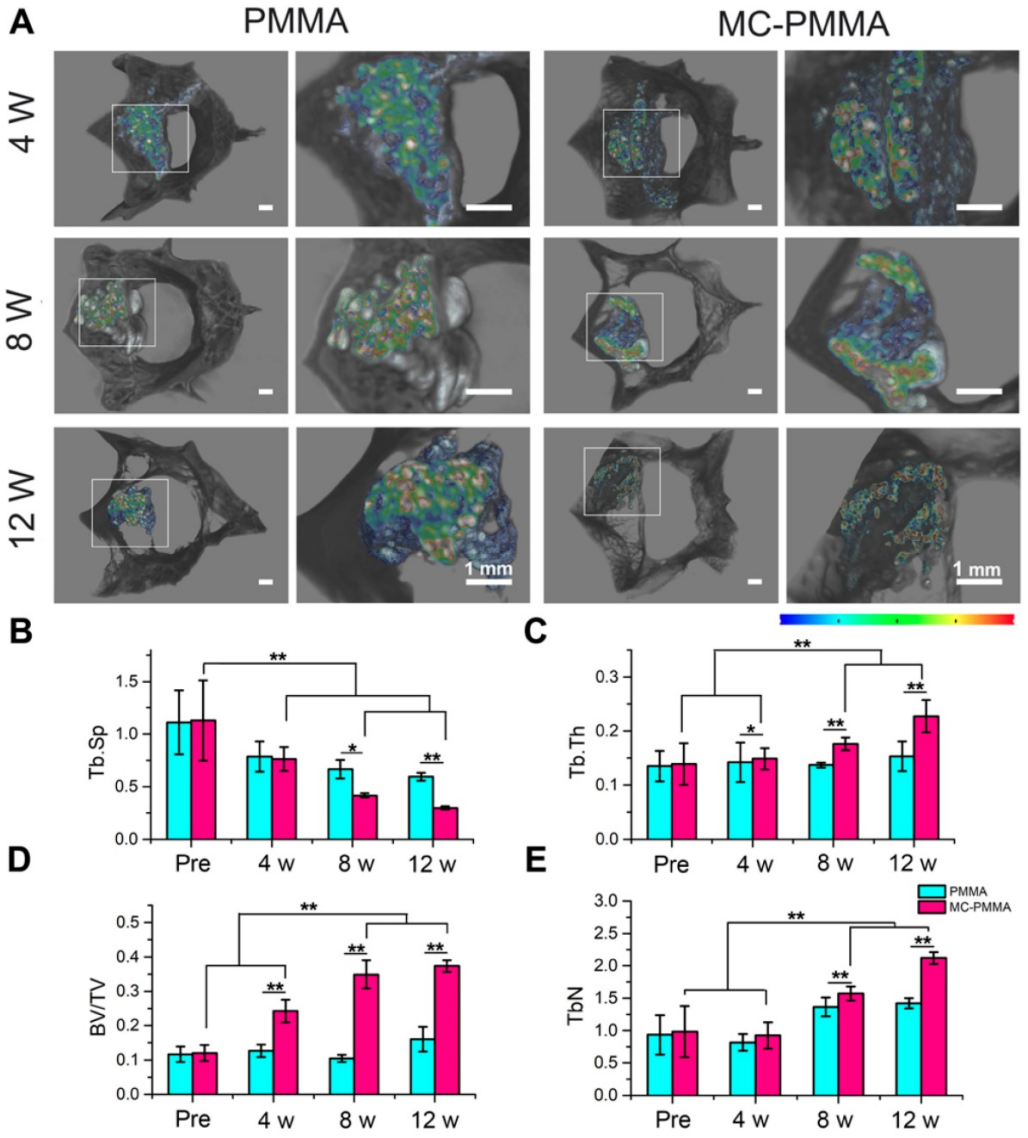
Micro-CT analysis of bone ingrowth in PMMA and MC-PMMA groups. (A) Reconstructed three-dimensional images of vertebral body with different CT values in different colors. (B) Trabecular separation (Tb.Sp, μm): the average width of the medullary cavity between the trabeculae, also known as trabecular space; an increased value means an increase in bone absorption. (C) Trabecular thickness (Tb.Th, μm): the average trabecular thickness in the selected area. (D) Bone volume fraction (BV/TV): the volume fraction of bone in the selected area, equal to the volume of bone trabeculae (BV) divided by the sample volume (TV). (E) Trabecular number (Tb.N, 1/mm): the number of intersections between bone and non-bone in the selected area. The CT values were 33237-44499. Results are presented as the mean ± SD; n = 6; *p < 0.05 and **p < 0.01.

X-ray and micro-CT images showed that both sets of specimens were firmly bonded to the host bone, with no obvious gaps. Three-dimensional rendering by micro-CT showed the location of the defect and bone cement. In Figure 7A, the bone cement has a high CT value shown in red and yellow, while the bone is in black. As the bone cement is replaced by bone, the color changes to green, blue, and finally black, indicating gradually lowered CT values. At 4 weeks, the CT value and volume of the bone cement were similar between the two groups of specimens. At 8 weeks, the CT value decreased in the MC-PMMA group, but was almost the same in the PMMA group. At 12 weeks, there were more areas with CT values similar to those of bone in the MC-PMMA group than before. However, the CT values of the PMMA group remained unchanged. The difference in the interfacial appearance and CT value of the cement indicated more material resorption and bone in growth in the MC-PMMA group than in the PMMA group. Quantitation of the reconstructed three-dimensional images of the vertebral body showed more bone formation in the MC-PMMA bone cement group than in the PMMA bone cement group at 4, 8, and 12 weeks post-surgery (Figure 7B-E). At 4 weeks post-surgery, there was a higher percentage of bone volume (BV/TV, 24.24 ± 3.27%, P=0.001, n=6) and higher trabecular thickness (Tb.N, 0.93 ± 0.21 mm^-1^, P=0.001, n=6) in the MC-PMMA group versus the control group (BV/TV, 12.67 ± 1.84%, Tb.N, 0.71 ± 0.12 mm^-1^, n=6). However, there was no significant difference in either trabecular thickness (Tb.Th, 0.14 ± 0.02 mm, Tb.Th, 0.15 ± 0.02 mm, P=0.599, n=6) or trabecular separation (Tb.Sp, 0.76 ± 0.11 mm^-1^, Tb.Sp, 0.78 ± 0.14 mm^-1^, P=0.683, n=6) between the specimens in the two groups. At 8 and 12 weeks post-surgery, a significantly higher trabecular thickness (Tb.Th, 0.17 ± 0.01 mm, 0.23 ± 0.03 mm, P=0.001, n=6), a higher percentage of bone volume (BV/TV, 34.89 ± 4.06%, 37.33 ± 1.65%, P=0.001, n=6), a higher trabecular number (Tb.N, 1.57 ± 0.11 mm^-1^, 2.12 ± 0.09 mm^-1^, P=0.001, n=6), and a lower trabecular separation (Tb.Sp, 0.41 ± 0.02 mm^-1^, 0.30 ± 0.01 mm^-1^, P=0.007, n=6) were found in the MC-PMMA group compared to that of the PMMA group, indicating increased bone growth in MC-PMMA group with time.

### Clinical study

#### Clinic effects of treatment with OVCFs

**Figure 8:**
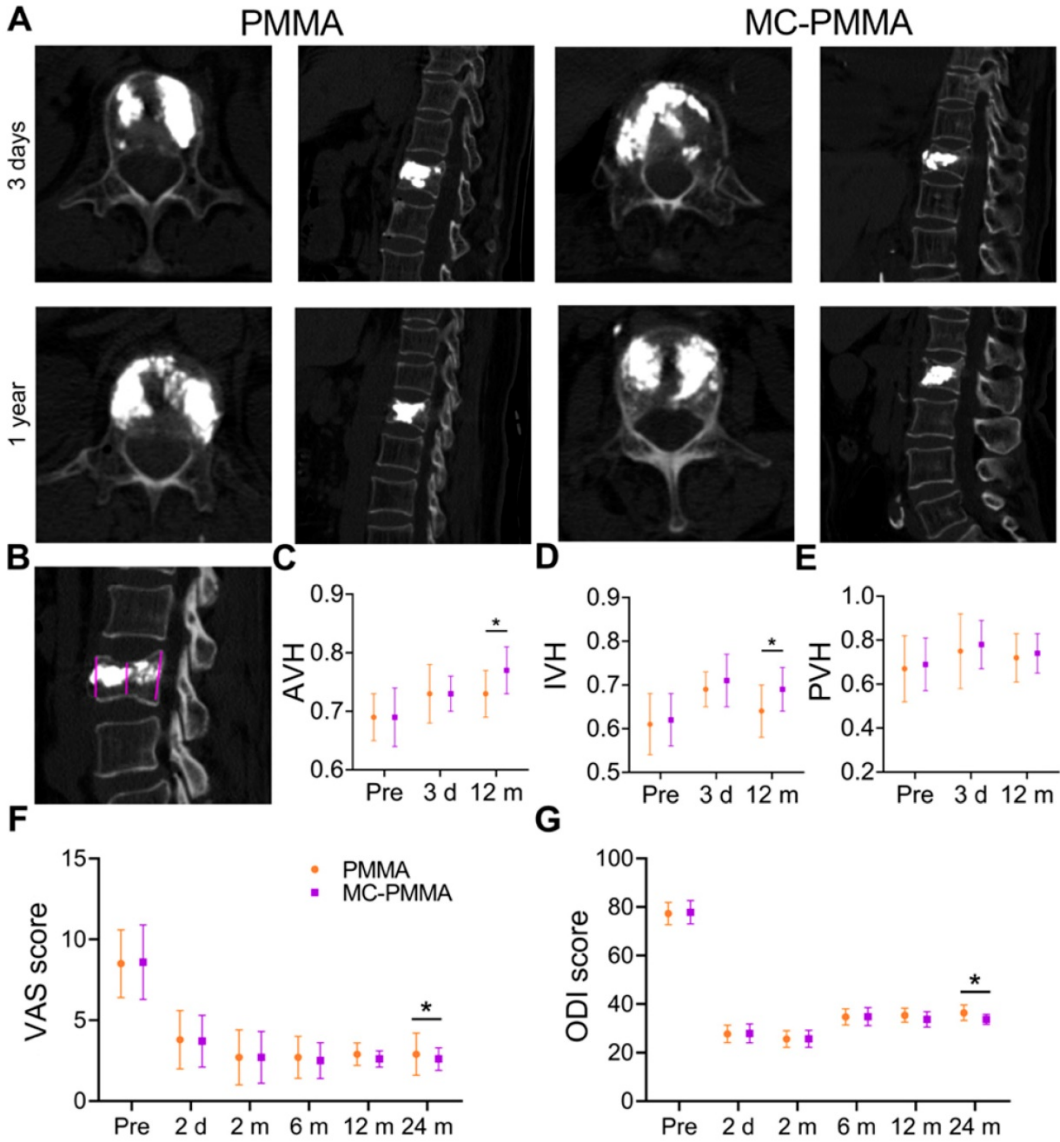
Clinical application of PMMA and MC-PMMA bone cements in OVCFs. (A) Lateral projection re-examination by CT at 3 days and 1 year postoperatively. (B) Pattern diagram of AVH, IVH, PVH. (C) AVH, mean anterior vertebral height of fractured vertebra/mean anterior vertebral height of the superjacent vertebra. (D) IVH, mean intermediate vertebral height of fractured vertebra/mean anterior vertebral height of the superjacent vertebra. (E) PVH, mean posterior vertebral height of fractured vertebra/mean anterior vertebral height of the superjacent vertebra. The VAS score (F) and ODI score (G) were evaluated by three doctors. Results are presented as the mean ± SD; *p < 0.05.

There were no postoperative infections or intraoperative embolic complications on the injection of the cement. None of the patients required transfusion, and the amount of bleeding was minimal (14.2 ± 3.4 mL). The images of patients after surgery were almost the same (Figure 8A). There was no statistically significant difference in sex, age, or bone mineral density between the patients in the two groups (Table S2). The number of recurrent fractures of adjacent vertebrae of MC-PMMA bone cement was 0, and that of PMMA bone cement was 8. The baseline patient diagnosis and treatment flowchart are shown in the Supplementary Materials. The anterior vertebral height (AVH) and intermediate vertebral height (IVH) were reduced after the fracture (Figure 8C‒D). Very little or no height was lost in the posterior wall(Figure 8E). The IVH was the lowest in both groups before surgery and changed significantly after surgery. The posterior vertebral height decreased slightly. Compared with those in the PMMA bone cement group, vertebral heights were almost the same before surgery and 3 days after the surgery in the MC-PMMA group. After 1 year and 2 years, there were significant differences between the two groups in AVH and IVH (Figure 8C‒E). There was no significant difference in either the Visual Analog Scale (VAS) or Oswestry Disability Index (ODI) values between the two groups after surgery (Figure 8F‒G). After 2 years, VAS and ODI values in the MC-PMMA group were significantly lower than those in the PMMA group (Figure 8F‒G). For the postoperative follow-ups, VAS and ODI were significantly improved compared with preoperative values in both the MC-PMMA and PMMA groups. The VAS scores of the MC-PMMA group decreased from 8.6 ± 2.3 to 2.8 ± 1.1, and those of the PMMA group decreased from 8.5 ± 2.1 to 2.9 ± 1.3. The ODI values of MC-PMMA group decreased from 77.8 ± 4.8 to 34.7 ± 2.1 and those of the PMMA group decreased from 77.3 ± 4.6 to 36.4 ± 3.2.

## Discussion

OVCFs caused by osteoporosis are common in the elderly population 6, 32. Clinical treatments of OVCFs include traditional therapies (such as oral medications and immobilization) and surgical treatment 33, 34. To improve curative effects and relieve patient suffering, vertebral augmentation/stabilization procedures, such as PVP and BKP, have been widely used in the treatment of OVCFs in recent years 15, 35. PMMA bone cement is universally used in vertebral prosthesis with two major drawbacks: a high elastic modulus and a lack of biological activity, which limit its application 36. MC is a biomimetic composite material that simulates the chemical composition and microstructure of the natural bone matrix 37, 38. Therefore, we added MC to a commercially available PMMA bone cement to improve its mechanical properties and biological activity, followed by its evaluation in cell studies, *in vivo* studies, and a small-scale clinical study.

Our research showed that adding a defined amount of MC to PMMA bone cement could maintain its operational characteristics and significantly reduce the mechanical properties of the original bone cement while maintaining its excellent properties. MC in the powder of PMMA absorbed a part of the liquid MMA and influenced the mixture of MMA and PMMA, which reduced the doughing and manipulation times. However, the addition of MC was not sufficient to produce statistical differences. Both the PMMA and MC-PMMA bone cement meet the clinical operational requirements of PVP and BKP. However, the introduction of MC particles changed the structure of the original PMMA in the process of PMMA polymerization, resulting in a decrease in the mechanical properties of the original PMMA. In this study, MC-PMMA bone cement had a reduced elastic modulus and compressive strength compared with PMMA bone cement. Bone cement with a high modulus could break the stress uniformity of the original vertebral body, causing stress concentration in bone tissue near the bone cement. When the stress of the bone trabeculae around the cement was large, the bone trabeculae were prone to fracture recurrence, leading to vertebral collapse. When the elastic modulus of bone cement was low, the stress concentration of the surrounding bone tissue caused by the bone cement was significantly reduced, which was conducive to the stability of the original vertebral body and provided implantation longevity 39, 40. Accumulating evidence has also suggested critical correlations between the modulus of bone cement and the risk of vertebral re-collapse after vertebroplasty 41. A previous *ex vivo* study on osteopenic human vertebrae also found a better effect on the improvement of vertebral stiffness using bone cement with low modulus 42. Robo et al. 18 found that linoleic acid (LA)-modified acrylic bone cement exhibited lower mechanical properties than traditional PMMA bone cement and a reduced elastic modulus of up to 65%, without changing the injectability and operating time of the PMMA bone cement. An *in vitro* biomechanical study by Boger et al. 43 demonstrated that the failure strength of augmented functional spine units could be better preserved using low-modulus PMMA, rather than regular PMMA cement. The spine is mainly composed of trabecular bone. The compressive strength of human trabecular bone is 2-12 MPa 44, 45, which is much lower than 80-120 MPa for PMMA bone cement 46. An excessively low compressive strength can lead to the deformation of the vertebral body under heavy pressure or impact. However, an excessively high compressive strength will cause a mismatch in stiffness between PMMA bone cement and the adjacent vertebral body, which can easily lead to a stress shielding effect, abnormal load transfer, and secondary fracture of adjacent vertebral bodies, especially in patients with osteoporosis 47. In our study, the compressive strength of MC-PMMA bone cement was significantly lower than that of PMMA bone cement; however, it was higher than the 70 MPa, indicating that the addition of MC could maintain a suitable compressive strength for treating osteoporosis vertebra. Therefore, MC-PMMA had better mechanical properties, alleviated the phenomenon of mechanical concentration, and supported the mechanical properties of the original vertebral body 48.

The biological activities of MC-PMMA bone cement were significantly improved compared with PMMA bone cement, as confirmed by *in vitro* and *in vivo* studies. Calcium phosphate mineral and collagen in MC is a critical component of a range of biomaterials for osteogenic repair applications 49-51. With the addition of MC, MC-PMMA bone cement could resist cell-mediated contraction and promote a more osteogenic phenotype. The *in vitro* results showed that MC-PMMA bone cement promoted the adhesion, proliferation, and osteogenic differentiation of BMSCs, which may benefit from the uniformly compounded calcium phosphate mineral and collagen and a similar ratio of Ca/P to the natural bone. Additionally, BMSCs grown on MC-PMMA bone cement exhibited stronger osteogenic differentiation and higher gene expression than those grown on PMMA bone cement, which was consistent with our previous microarray results, indicating that MCs could upregulate osteogenesis-related genes, such as BMP-2 and OPN, and stimulate osteogenic differentiation through the osteoblast differentiation and skeletal system development pathways 52. After implantation, the lack of biological activity of PMMA bone cement impedes its integration with the adjacent bone. At 12 weeks, the interface between the implanted PMMA and the skeleton remains intact, and the material cannot combine with the autogenous skeleton (Figure 6). Additionally, due to the compactness and non-biodegradation of the PMMA bone cement, the defect area was filled with little fibrous tissue, and no new bone formation was observed in the PMMA group (Figure 7). However, micro-CT analysis demonstrated that the MC-PMMA bone cement fused well with the host bone. The MC-PMMA bone cement could combine with the surrounding bone tissue because of its biological activity and biodegradability, which is conducive to reducing the risk of cement loosening or shedding and improving the safety of bone cement. First, the MC-PMMA bone cement had a biocompatible surface that could promote cell adhesion and proliferation, which was beneficial for recruiting osteoblasts and promoting cell migration *in vivo*. Second, the MC particles in the modified bone cement were gradually degraded and absorbed, forming pore structures on the surface of the bone cement. The degradation of MC was conducive to guiding new bone tissue to grow into the porous structure, thus forming an interlaced mosaic and occlusion between the bone cement and bone tissue at the implantation site. At the same time, the internal MC particles would contact in the way of bridging, promoting osteogenesis to gradually crawl from the surface to the interior of PMMA. Additionally because of the irregularity of trabecular bone and compression fracture defect, the injected MC-PMMA showed a dendritic shape and large specific surface area. More MC particles were exposed at the interface with the surrounding bone, which was very beneficial for the recovery of the original vertebral body and bone regeneration. Moreover, the longevity of the remaining PMMA bone cement could provide extended mechanical support to the vertebral body, maintaining good mechanical properties, which is beneficial to the stability of the bone cement and reduces the risk of loosening or even slipping.

In previous animal model studies of PVP, large animals, such as sheep and dogs, were used 53-55. Oliveira et al. used PVP surgery in merino sheep at a cost of US$600,000-650,000 56. Lu et al. subjected four healthy mongrel dogs to PVP under CT guidance 57. Yang et al. used PVP with interstitial implantation of 125I seeds, and 125I seeds were transplanted into the vertebral body at the T13 level of the spine in banna mini-pigs 58. In the present study, we established a rabbit model of osteoporosis for PVP surgery. Accordingly, we established a more comprehensive model of osteoporosis through castration combined with glucocorticoid injections 59-61. Compared to other PVP animal models in the literature, our rabbit model in this study requires less time and has lower costs. In our model, the mortality rate was low, and the model results were relatively reproducible. Our model achieved bone loss and remodeling in mature mammals. Subsequently, we injected bone cement into the vertebral body of the osteoporotic rabbits under the guidance of X-ray through a developed push rod. This surgery effectively simulates PVP surgery in humans, and MC-PMMA and PMMA bone cements can be injected into the rabbit vertebral body. During the surgery, we needed to make an incision in the rabbit's skin. We simply needed to insert the needle directly through the skin and inject our material into the vertebral body with little or no bleeding. The material can exist in rabbits for 8 weeks or more, and its properties can be evaluated thoroughly. Thus, we achieved safe and effective PVP in a small animal model.

The small-scale clinic study paved the way for a large number of clinical applications. One complication of PVP and BKP is re-fracturing of the adjacent vertebral body. Hulme et al. 62 reviewed 69 clinical series on BKP and reported a re-fracture rate of 15%, and most were at adjacent levels. Han et al. 63 reviewed 47 clinical series on PVP and reported 29.8%. In the present study, the re-fracture rate in the PMMA bone cement group was similar to that reported in the literature, and there were no re-fractures in the MC-PMMA group. In both groups of patients, at a follow-up of 2 years, both VAS and ODI values decreased significantly after PVP. At 1 and 2 years after surgery, the ODI and VAS values in the MC-PMMA group were lower than that of the PMMA group, indicating that the MC-PMMA bone cement had a better long-term effect. In both groups, the vertebral height of patients decreased after 2 years. The degree of decrease in vertebral height in the MC-PMMA group was significantly smaller than that in the PMMA group, which explained the better effect of MC-PMMA bone cement on preventing recompression.

Despite our promising findings, further work on clinical studies is needed by, for example, increasing the number of cases and the length of follow-up in more hospitals and clinics. Additional examinations of the patients should be performed. Further multicenter studies in other hospitals and research centers should be carried out to better corroborate our conclusions.

## Conclusions

We developed bioactive PMMA-based bone cement by adding mineralized collagen to the powder of a commercially available PMMA bone cement brand that is widely used in PVP and BKP (“MC-PMMA bone cement”). Compared to PMMA bone cement, the compressive modulus of MC-PMMA bone cement was significantly lower, while handling times were approximately the same. MC-PMMA bone cement facilitated cell proliferation and differentiation and accelerated the repair of vertebrae in an osteoporotic rabbit model and OVCFs in patients in a small-scale clinical test. Our results show that MC-PMMA bone cement is promising for clinical transformation.

## Figures and Tables

**Table 1:** Mixing compositions of MC-PMMA bone cement

Sample	Powder(g)				Liquid(ml)		
	MC	Poly(methyl methacrylate-*co*-methyl acrylate)	BaSO_4_/ZrO_2_	Benzoyl peroxide	Methyl methacrylate	N,N-dimethyl-*p*-toluidine	Hydroquinone
Mendec Spine(PMMA)	0	13.5	6.0	0.5	9.91	0.09	75ppm
MC-Mendec Spine(PMMA)	3	13.5	6.0	0.5	9.91	0.09	75ppm

**Table 2:** Histological scores.

Tissue response at the bone-material interface	Score
Direct bone to implant contact	4
Remodeling lacunae with osteoblasts/osteoclasts	3
Mainly fibrous tissue capsule formation	2
Unorganized fibrous tissue	1
Inflammation with inflammatory cells/unorganized tissue	0
New bone quality	Score
Almost compact bone	3
Thick cortical bone surrounded with dense trabeculae	2
Thin dense bones surrounded with dense trabeculae	1
Thin layer of dense bone surrounded with loose trabecula	0
Micro-CT and Histology of bone ingrowth	Score
Bone ingrowth over entire defect	3
Bone ingrowth over part of defect	2
Bone growth only at defect borders	1
No bone formation within defect	0

## Abbreviations

ALP: alkaline phosphatase
ANOVA: analysis of variance
AVH: anterior vertebral height
BMD: bone mineral density
BMP2: bone morphogenetic protein-2
BMSCs: bone mesenchymal stem cells
BSA: bovine serum albumin
DMEM: Dulbecco's modified Eagle medium
ECM: extracellular matrix
EDTA: ethylene diamine tetra acetic acid
FBS: fetal bovine serum
FTIR: Fourier Transform Infrared Spectrometer
HA: hydroxyapatite
IVH: intermediate vertebral height
MC: mineralized collagen
micro-CT: microcomputed tomography
OCN: osteocalcin
ODI: Oswestry disability index
OPN: osteopontin
OVCFs: osteoporotic vertebral compression fractures
PBS: phosphate buffered saline
PMMA: poly(methyl methacrylate)
PVP: percutaneous vertebroplasty
qRT-PCR: quantitative real-time polymerase chain reaction
Runx2: runt-related transcription factor 2
SD: standard deviation
SEM: scanning electron microscopy
VAS: visual analog scale
