# Supplementary material for: Bioactive poly (methyl methacrylate) bone cement for the treatment of osteoporotic vertebral compression fractures
Source: Theranostics. 2020 May 17;10(14):6544–60. doi: 10.7150/thno.44428 (PMC7255031; doi:10.7150/thno.44428)
Supplement: Supplementary file 1 — Supplementary figures and tables. [file thnov10p6544s1.pdf]

Supporting Information

Bioactive poly (methyl methacrylate) bone cement for the treatment of osteoporotic vertebral compression fractures

Jinjin Zhu[1,2,3†], Shuhui Yang[1†], Kaiwen Cai[3], Shuo Wang[1], Zhiye Qiu[1], Junfei Huang[4], Guoqiang Jiang[3*], Xiumei Wang[1*], Xiangqian Fang[2*]

1. State Key Laboratory of New Ceramics and Fine Processing, School of Materials Science and Engineering, Tsinghua University, Beijing 100084, China
2. Department of Orthopaedic Surgery, Sir Run Run Shaw Hospital, Zhejiang University School of Medicine & Key Laboratory of Musculoskeletal System Degeneration and Regeneration Translational Research of Zhejiang, Hangzhou 310016, China
3. Department of Spinal Surgery, The Affiliated Hospital of Medical School of Ningbo University, Ningbo 315020, China
4. Shimadzu (China) Co., Ltd. Shenzhen Branch, Shenzhen 518042, China

*Corresponding author: Xiumei Wang, E-mail: wxm@mail.tsinghua.edu.cn, Phone: 86-10-62782966. Fax: 86-10-62771160. Guoqiang Jiang, E-mail: jgq6424@hotmail.com. Xiangqian Fang, E-mail: orthofxq@zju.edu.cn.

†These authors contributed equally to this work.

Methods

Porosity determination

Each of the bone cement samples (Φ 13 mm × 1 mm, n = 5) were scanned by Micro-CT. Each sample was then reconstructed using a data analyzer VGStudio under the same condition. An industrial micro-CT system was used at 225 kV accelerating voltage and 4-μm resolution to scan the vertebral body. The images of cylinders (1.5 mm diameter × 1.5 mm height) in the bone cement samples were shown in Figure S1 A-D. The porosities of bone cements were statistically analyzed using Image Pro Plus, as shown in Figure S1E and F.

Clinical applications

General information

This study was approved by the Ethics Committee of the Affiliated Hospital of Medical School of Ningbo University (2015KY0903). From July 2016 to July 2018, 24 patients were diagnosed with single vertebral fractures with osteoporosis, including T11, T12, L1, L2, L3, and L4 vertebral fractures. Preoperative vertebral X-rays (Toshiba X-ray machine; Toshiba, Tokyo, Japan), magnetic resonance imaging (Signa VH/i 1.5T; General Electric Company, New York, USA), and CT examinations (Philips iCT; Philips Medical System, Amsterdam, Netherlands) were performed for each patient. Patients who experienced spinal stenosis, disc disease, and vertebral lesions caused by the dural sac and nerve root compression causing pain were excluded. Each of the patients had mild to severe back pain and was refractory to conservative treatment for at least 4 weeks. The patients were randomly divided into two equal-sized groups (Scheme 1D). The clinical data and BMD of patients were assessed from L1 to L4 and the hip joint by dual-energy X-ray absorptiometry (DISCOVERY CT750 HD; General Electric Company, New York, USA).

Surgical techniques

The surgical procedure was the same as that reported previously [64]. The puncture route was clearly defined by a puncture needle in the coronal plane: the upper quadrant of the bilateral pedicles (approximately 10 o'clock in the left and 2 o'clock in the right).

The angle of the needle to the bilateral pedicle was approximately 12–18° on the horizontal plane. A puncture channel was established. Subsequently, a balloon was inserted into the fractured vertebral body to restore the height and create a cavity in the vertebral body. Then, dough of the bone cement was slowly injected into the fractured vertebral body. All patients were placed on bed rest for 24 h after surgery.

Clinical and radiological evaluations

The clinical evaluation involved the determination of the Visual Analog Scale (VAS) score to evaluate of pain (from 0 [no pain] to 10 [most pain]) and the Oswestry Disability Index (ODI) score for the functional assessment of patients at 3 days, 3 months, 6 months, 1 year and 2 years post-operation. Digitized images and CT values were analyzed using Spineview software by two independent radiology clinicians who were not otherwise involved in this study. CT imaging included the following: (1) standard radiography of the thoracolumbar spine while standing; (2) systematic CT scans (after surgery, 3 days and 2 years postoperatively).

Table S1. Primer sequences used for quantitative RT-PCR.

Gene	Primers(F=forward, R=reverse)	Amplicon(bp)
BMP2	F：GAAGCCAGGTGTCTCCAAGAG	142
	R：GTGGATGTCCTTTACCGTCGT	
ALP	F：AGCGACACGGACAAGAAGC	183
	R：GGCAAAGACCGCCACATC	
Runx2	F：CAGACCAGCAGCACTCCATA	256
	R：CAGCGTCAACACCATCATTC	
OCN	F：AGATTGTTGGGGCACAAGGT	126
	R：CCTTCAGCAGGGAAACCGAT	
OPN	F：GGAGTCCGATGAGGCTATCAA	208
	R：TCCGACTGCTCAGTGCTCTC	

Table S2. Patients' Clinical Data

Parameters	Group MC-PMMA	Group PMMA
Number of patients	12	12
Mean age (year)	74.12 ± 9.31	75.31 ± 8.77
Sex (male/female)	9/3	7/5
BMD of L1-L4 (T value)	3.01 ± 1.21	2.98 ± 1.47
Recurrent fracture	0	8

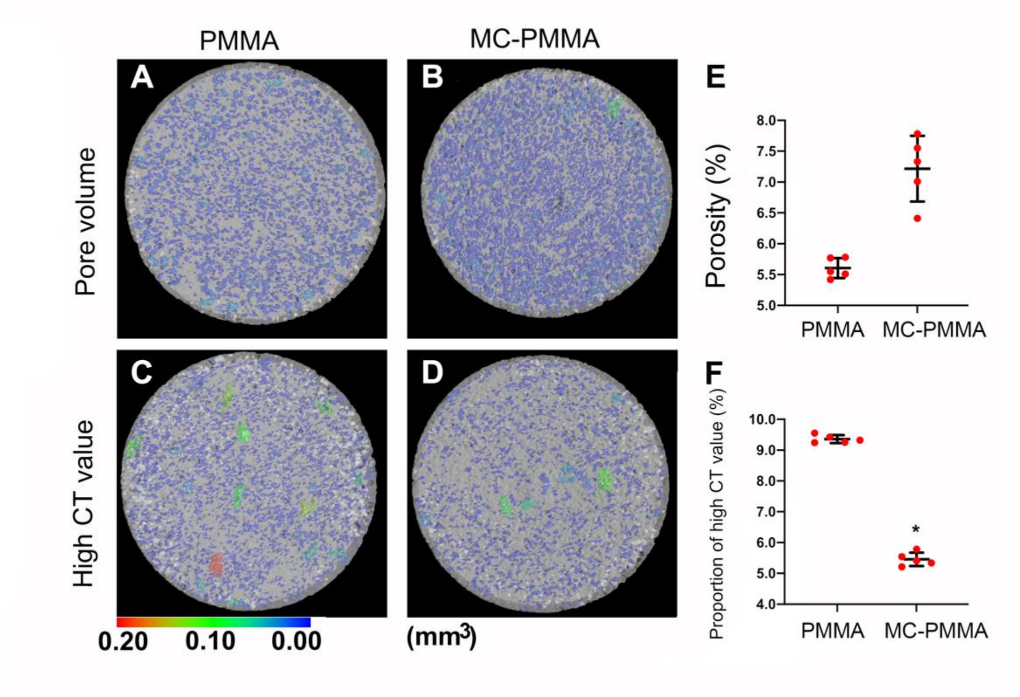

Figures S1. (A, B) Pore volume of bone cement. (C, D) High CT value of bone cement. (E) Porosity of bone cement. (F) Proportion of high CT value of bone cement. Values are represented as mean ± SD. n = 5, *p < 0.05.
